# Bone morphogenetic protein signaling regulation of AMPK and PI3K in lung cancer cells and *C. elegans*

**DOI:** 10.1186/s13578-022-00817-3

**Published:** 2022-05-31

**Authors:** Mehul Vora, Arindam Mondal, Dongxuan Jia, Pranya Gaddipati, Moumen Akel, John Gilleran, Jacques Roberge, Christopher Rongo, John Langenfeld

**Affiliations:** 1 Department of Surgery, Rutgers Robert Wood Johnson Medical School, Rutgers, The State University of New Jersey, New Brunswick, NJ 08903 USA; 2 Department of Genetics, The Waksman Institute, Rutgers the State University of NJ, Piscataway, NJ 08854 USA; 3 Rutgers University, Piscataway, NJ 08854 USA; 4 Molecular Design and Synthesis, RUBRIC, Office for Research, Rutgers Translational Science, Rutgers University, Piscataway, NJ 08854 USA

**Keywords:** BMP, AMPK, PIK3, Akt, mTOR, Lung cancer, Metabolism, LKB1, BMPR2

## Abstract

**Background:**

Bone morphogenetic protein (BMP) is a phylogenetically conserved signaling pathway required for development that is aberrantly expressed in several age-related diseases including cancer, Alzheimer’s disease, obesity, and cardiovascular disease. Aberrant BMP signaling in mice leads to obesity, suggesting it may alter normal metabolism. The role of BMP signaling regulating cancer metabolism is not known.

**Methods:**

To examine BMP regulation of metabolism, *C. elegans* harboring BMP gain-of-function (*gof*) and loss-of-function (*lof*) mutations were examined for changes in activity of catabolic and anabolic metabolism utilizing Western blot analysis and fluorescent reporters. AMP activated kinase (AMPK) *gof* and *lof* mutants were used to examine AMPK regulation of BMP signaling. H1299 (LKB1 wild-type), A549 (LKB1 *lof*), and A549-LKB1 (LKB1 restored) lung cancer cell lines were used to study BMP regulation of catabolic and anabolic metabolism. Studies were done using recombinant BMP ligands to activate BMP signaling, and BMP receptor specific inhibitors and siRNA to inhibit signaling.

**Results:**

BMP signaling in both *C. elegans* and cancer cells is responsive to nutrient conditions. In both *C. elegans* and lung cancer cell lines BMP suppressed AMPK, the master regulator of catabolism, while activating PI3K, a regulator of anabolism. In lung cancer cells, inhibition of BMP signaling by siRNA or small molecules increased AMPK activity, and this increase was mediated by activation of LKB1. BMP2 ligand suppressed AMPK activation during starvation. BMP2 ligand decreased expression of TCA cycle intermediates and non-essential amino acids in H1299 cells. Furthermore, we show that BMP activation of PI3K is mediated through BMP type II receptor. We also observed feedback signaling, as AMPK suppressed BMP signaling, whereas PI3K increased BMP signaling.

**Conclusion:**

These studies show that BMP signaling suppresses catabolic metabolism and stimulates anabolic metabolism. We identified feedback mechanisms where catabolic induced signaling mediated by AMPK negatively regulates BMP signaling, whereas anabolic signaling produces a positive feedback regulation of BMP signing through Akt. These mechanisms were conserved in both lung cancer cells and *C. elegans*. These studies suggest that aberrant BMP signaling causes dysregulation of metabolism that is a potential mechanism by which BMP promotes survival of cancer cells.

## Background

Bone morphogenetic proteins (BMP) are phylogenetically conserved, orchestrating essential developmental processes from metazoans to mammals [1]. BMP signaling is increased in several age-related diseases including obesity [2], Alzheimer’s disease [3–6], cardiovascular disease [7] and cancer [8, 9]. Although a causative role for aberrant BMP activity in age-related diseases has been reported, a common mechanism has not been established.

There are over 20 BMP ligands that are divided into four distinct subtypes based on structure similarity and function [10]. BMP ligands signal through receptor serine/threonine kinases. BMP ligands bind to type 1 BMP receptors (BMPR1) (ALK2, ALK3, or ALK6) promoting phosphorylation by the constitutively active BMP type 2 receptors (BMPR2) (BMPR2, ActR-IIA, ActRIB) [11], which then phosphorylate Smad- 1/5, inducing transcriptional activation of downstream target genes. Transcriptional targets of BMPR1-Smad-1/5 include the inhibitor of differentiation proteins (ID1-ID4), which regulate survival, migration, proliferation, and self-renewal of stem cells and cancer cells [12, 13]. BMP2/4 and BMP7 also signal through BMPR2 without activating Smad-1/5 signaling. BMPR2 Smad-independent signaling includes an upregulation of anti-apoptotic proteins, X chromosome-linked inhibitor of apoptosis protein (XIAP) and transforming growth factor beta (TGFβ) activated kinase 1 (TAK1), which is also mediated in both embryonic development and survival of cancer cells [14–16].

Genetic and evolutionary analysis suggests that the increased expression of BMPR2 mRNA in adipose tissue of obese patients has been selected because it is a metabolic “thrifty” gene that is contributing to present day obesity [17]. Recent studies in mice showed that enhanced BMP activity causes obesity, hyperglycemia, and insulin resistance [18, 19]. The mechanism by which overactive BMP signaling contributes to obesity is not known. BMP ligands have been shown to activate PI3K during development as well as to induce cell migration [20–23]. PI3K promotes fat production by activating Akt, which promotes lipid and fatty acid synthesis [24]. Since BMP has had a significant developmental role for over 700 million years [25], and its overexpression promotes obesity in humans, we addressed whether BMP signaling has a broader role in cellular metabolism.

Utilizing lung cancer cells that have aberrant BMP signaling and *C. elegans*, we examined BMP signaling for a role in catabolic and anabolic metabolism. We find that in addition to promoting anabolic signaling through BMPR2 activation of PI3K, BMP signaling also suppresses AMP activated kinase (AMPK), the master regulator of catabolism. Furthermore, feed-back regulation by AMPK suppresses BMP signaling, whereas feed-forward regulation by Akt enhances it. BMP2 ligand suppressed expression of tricarboxylic acid cycle (TCA) intermediates and non-essential amino acids. These studies provide evidence that aberrant BMP signaling alters normal metabolic signaling events, which has implications as a potential contributing cause of the metabolic dysfunction in cancer and other age-related diseases.

## Methods

### General methods and strains

All *C. elegans* strains are derived from the Bristol strain N2 [26] and were grown at 20 °C on standard nematode growth media plates seeded with OP50 *E. coli*. Nematode cultures, genetic crosses, and other *C. elegans* husbandry were performed according to standard protocols [26].

### Imaging

*C. elegans* at L4 stage were mounted on 2.5% agarose pads with 10 µM Levamisole. Fluorescence images were captured on a standard epifluorescent microscope. Quantification was carried out using the Fiji suite [27]. At least 30 animals per condition/genotype/treatment were used for quantification and statistics.

### RNA isolation and qRT-PCR

Total RNA was extracted from *C. elegans* at 48 h after the L4 stage. *C. elegans* were synchronized by bleaching [28]. Total RNA was extracted by the freeze cracking method as previously described [33]. After RNA isolation, 2 µg of total RNA was primed with oligo(dT) and reverse transcribed to yield cDNA using the SuperScript III reverse transcriptase kit as per manufacturer’s protocol (Invitrogen). Real-time PCR was performed on QuantStudio3 (Applied Biosystems by Thermo Fisher Scientific) instrument using the PowerUP SYBR Green master mix (Applied Biosystems) according to manufacturer’s instructions. The experiments were performed in three technical replicates for each condition. Primer sequences for *nhr-49* were forward primer TCCGAGTTCATTCTCGACG and reverse primer GGATGAATTGCCAATGGAGC [29]. Primer sequences for *spp-9* were forward primer GTTCTCTTTCTGGTTGCGGT and reverse primer GCTCTACAAACATCTTCTGGTGCA. Primer sequences for *act-1* were forward primer CCATCATGAAGTGCGACATTG and reverse primer CATGGTTGATGGGGCAAGAG [30]. QuantStudio Design and Analysis Software v1.5.1 was used to calculate raw Ct values and to normalize the values for *dbl-1* and *spp-9* to the housekeeping gene *act-1* (Actin) (Applied Biosystems by Thermo Fisher Scientific). Fold-change in gene expression between experimental sample and the wild-type control was determined by this software using the formula: 2^(−ΔΔCt)^. Experimental ΔCt values were compared with wild-type ΔCt values using the unpaired *t* test.

### Western blotting and quantification

*C. elegans* were synchronized by alkaline bleaching and arrested at L1 stage on unseeded NGM plates overnight. Arrested L1s were transferred to seeded plates and grown on standard NGM plates until L4 stage at 20 °C. Fifty synchronized L4 stage nematodes for each strain were placed in 15 μl M9 buffer and 15 μl NextGel protein sample loading buffer (4x) (VWR, catalog # M260-5.0 ML) was added, flash frozen in liquid nitrogen and stored at − 80 °C until used for western blotting. Protein samples were boiled for 5 min with sample buffer (Bio-Rad), then centrifuged at 13,000 rpm for 1 min. Western blot analysis were performed as previously reported [8]. Briefly, proteins from cells were extracted using RIPA buffer, protein determined using BCA method, separated on polyacrylamide gel by SDS-PAGE and transferred to nitrocellulose. Primary antibodies were rabbit monoclonal anti-ID1 (Calbioreagents, San Mateo, CA), rabbit anti-Actin, an affinity isolated antigen specific antibody (Sigma, Saint Louis, MO), rabbit monoclonal anti-pAMPKα (T173), rabbit monoclonal AMPKα, rabbit monoclonal pLKB1 (Ser428), rabbit monoclonal LKB1, rabbit BMPR2, rabbit monoclonal pACC (Ser79), rabbit monoclonal pAkt (S473), rabbit monoclonal Akt, rabbit monoclonal p-p70S6 kinase (Thr389), rabbit monoclonal p70S6 (Cell Signaling). Mouse anti-actin, Clone C4 was for Western blot analysis in *C. elegans* studies (Sigma-Aldrich, MAB1501). Quantification of band intensity was measured using ImageJ Software (NIH) and statistical comparisons were made using one-way ANOVA.

### Cell culture and reagents

A549 and H1299 cell lines obtained from patients with non-small cell lung cancer (ATCC) were cultured in Dulbecco’s modified Eagle’s medium (DMEM, Sigma Aldrich, Saint Louis, MO, USA) with 5% fetal bovine serum. Normal Human Primary Bronchial/Tracheal Epithelial Cells were from ATCC (PCS-300-010) and cultured in Airway Epithelial Cell Basal Medium (PCS-300-030) supplemented with Bronchial Epithelial Cell Growth Kit (PCS-300-040). JL5 was synthesized by John Gilleran and Jacques Roberge from Rutgers Molecular Design and Synthesis, and DMH1 (S7146) was purchased from Selleckchem. AICAR (A9978) was purchased from Sigma-Aldrich.

### Immunofluorescence imaging of lung cancer cells

**Fig. 1:**
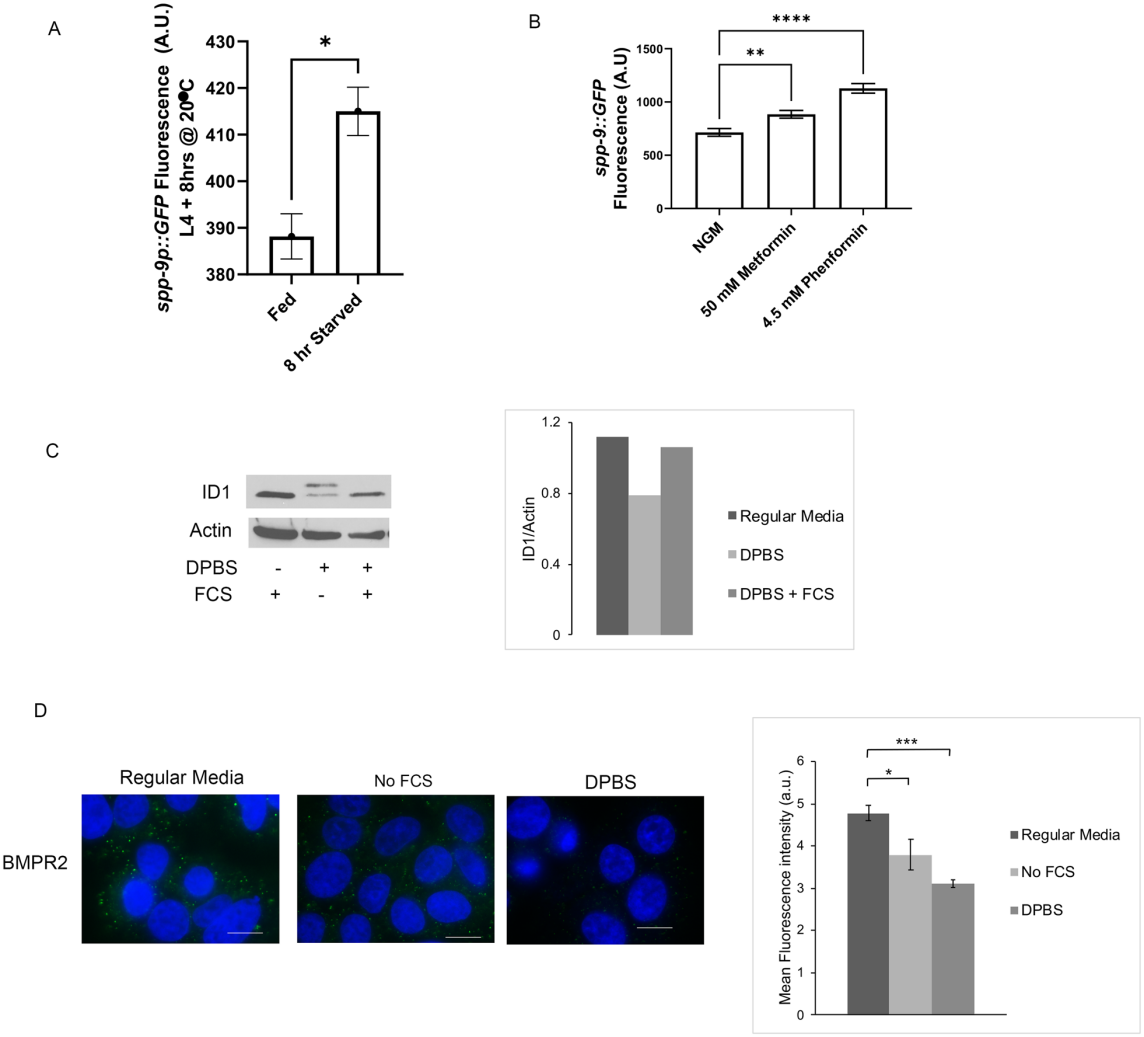
Nutrient stress suppresses BMP signaling. **A** Fluorescence quantification of *C. elegans* expression BMP transcriptional reporter *spp-9::GFP* after 8 h of starvation (n = 30 animals). **B** Fluorescence quantification of *C. elegans* BMP transcriptional reporter *spp-9::GFP* treated with 50 and 4.5 µM Metformin and Phenformin, respectively (n = 30 animals). Drugs were included in media for 3 days post-egg laying. **C** Western blot analysis of starved A549 cells with quantification. **D** Immunofluorescence image and quantification for BMPR2 of starved A549 cells. FCS indicates fetal calf serum. DPBS indicates Dulbecco’s phosphate buffered saline

Immunofluorescence imaging was performed as previously described [31]. Briefly, cells were plated onto cover slips and the next day treated for the designated time and washed. For starvation studies, cells were incubated overnight with regular media without fetal calf serum (FCS, R&D Systems # S11550) then incubated in DPBS with glucose (Gibco # 14,287,080) for 120 min (Fig. 1). In studies using human insulin (100 ng/ml, Sigma-Aldrich # I9278) treatment began 20 min after adding Dulbecco’s phosphate-buffered saline (DPBS) and continued for 60 min. MK-2206 (1 μM, Selleckchem # S1078) was give 1 h. prior to adding insulin. Cells were then stained with polyclonal rabbit anti-BMPR2 antibody (Sigma-Aldrich # HPA049014), which recognizes an extracellular epitope (Sigma-Aldrich). Cells were washed then incubated Alexa Flour 488 conjugated secondary antibody (Invitrogen # A-11008) for 1 h at room temperature. Cells were then washed with PBS and counterstained with DAPI (Sigma-Aldrich) for 10 min. Cells were then examined using 60X oil lens using a fluorescence microscope (Nikon eclipse TE300). Studies were performed at least 4 times.

### Treating lung cancer cells the BMP2

For studies examining response to BMP2 ligand (R&D Systems # 355-BM), lung cancer cells were incubated overnight with media without FCS. In the morning media was replaced with fresh media without FCS then treated with recombinant BMP2 (20 ng/ml) for designated times. In starved experiments, DPBS was added in the morning for 20 min then treated with BMP2 (20 ng/ml) or media with FCS for 20 min (Fig. 6C). In starved studies (Fig. 3G), following overnight incubation without FCS, cells were placed in DPBS cells and immediately treated with BMP2.

Lung cancer cells treated with insulin were incubated with media overnight without FCS, starved with DPBS for 20 min then treated with insulin (100 ng/ml) for 60 min (Fig. 6A, B). Cell treated with the Akt inhibitor MK-2206. MK-2206 were pre-treated 1 h. prior to adding insulin. Starvation studies using ligands were performed 3 times.

### Transient knockdown

Validated siRNAs (Life Technologies, ID: s2044) were used for BMPR2 knockdown. Negative control was Silencer Select negative control siRNA (4,390,843). H1299 lung cancer cells were transfected with siRNA using Lipofectamine® RNAiMAX Reagent (Invitrogen, Carlsbad, CA, USA). 750,000 cells were plated in a 6 well plate and were grown overnight. Next morning the cells were transfected with 6 nM BMPR2 or 6 nM of siRNA control for 48 h. according to manufacturer’s protocol. Knockdown experiments were performed 4 times.

### Metabolomic analysis by LC–MS

H1299 and A549 cells were incubated overnight in media without FCS. Cells were then treated with DMSO or BMP2 20 ng/ml for 40 min in triplicate. Cells were harvested with a 40:40:20 buffer of methano: acetonitrile: water and 0.5% formic acid. LC − MS analysis of the cellular metabolites was performed on the Q Exactive PLUS hybrid quadrupole-orbitrap mass spectrometer (Thermo Scientific) coupled to hydrophilic interaction chromatography (HILIC) as previously reported [32, 33]. The LC separation was performed on UltiMate 3000 UHPLC system with an XBridge BEH Amide column (150 mm × 2.1 mm, 2.5 μM particle size, Waters, Milford, MA) with the corresponding XP VanGuard Cartridge. The liquid chromatography used a gradient of solvent A (95%:5% H2O: acetonitrile with 20 mM ammonium acetate, 20 mM ammonium hydroxide, pH 9.4), and solvent B (20%:80% H2O: acetonitrile with 20 mM ammonium acetate, 20 mM ammonium hydroxide, pH 9.4). The gradient was 0 min, 100% B; 3 min, 100% B; 3.2 min, 90% B; 6.2 min, 90% B; 6.5 min, 80% B; 10.5 min, 80% B; 10.7 min, 70% B; 13.5 min, 70% B; 13.7 min, 45% B; 16 min, 45% B; 16.5 min, 100% B. The flow rate was 300 μl/min. Injection volume was 5 μL and column temperature 25 °C. The MS scans were in negative ion mode with a resolution of 70,000 at m/z 200. The automatic gain control (AGC) target was 3 × 106 and the scan range was 75 − 1000. Metabolite features were extracted in MAVEN [34].

### Statistical analysis

In lung cancer studies, paired student t-test assuming unequal variances was used to compare means. The mean of control was compared with the mean of each treated group. Differences with p values < 0.05 were considered statistically significant. The following signified *p < 0.05, **p ≤ 0.01, ***p ≤ 0.001, ****p ≤ 0.0001.

## Results

### Nutrient stress suppresses BMP signaling

To identify whether nutrient stress regulates BMP signaling, we subjected *C. elegans* to two different nutrient stress paradigms and monitored the expression of a BMP-regulated gene, *spp-9 *[35]. This gene is negatively regulated by BMP signaling, and thus an increase in the GFP signal of a *spp-9p::GFP* transcriptional reporter would indicate suppression of BMP signaling [35–37]. We find that starvation of *C. elegans* for 8 h leads to a decrease in BMP signaling (increase of SPP-9::GFP, Fig. 1A). Metformin and phenformin have been shown to induce a dietary restriction-like physiology in *C. elegans* [38], and we tested the effect of these treatments on BMP signaling. We find that both metformin- and phenformin-dependent nutrient stresses decrease BMP signaling (Fig. 1B). Importantly, this regulation of BMP signaling by nutrient deprivation is conserved in humans. A549 lung cancer cells exhibit a marked decrease in expression of ID1, a transcriptional target of BMP signaling, whereas ID1 levels are restored on addition of nutrients post-starvation (Fig. 1C).

Work in *C. elegans* has previously identified receptor trafficking as a mechanism by which BMP signaling might be regulated. Recycling of DAF-4, the nematode BMPR2, from the plasma membrane back to the surface after internalization is regulated via the recycling endosome, and BMPR2/DAF-4 levels are controlled by degradation within the lysosome [39]. We find that reduced nutrients by removal of fetal calf serum (FCS) decreased BMPR2 levels at the plasma membrane, and that this decrease was exacerbated by complete nutrient starvation (Fig. 1D).

Taken together, these studies demonstrate the conserved regulation of BMP signaling by nutrient stresses.

### BMP signaling suppresses the energy sensor and regulator AMPK in *C. elegans*

AMP Associated Kinase (AMPK) is a conserved, central regulator of nutrient sensing and energy homeostasis in many organisms. In *C. elegans*, AMPK, encoded by the *aak-2* gene*,* has a critical role in regulating the physiological response to metformin and phenformin, as well as to other dietary restriction paradigms [38, 40]. Upon nutrient stress, AMPK is activated by phosphorylation at Thr172 (humans) [41] and Thr243 (*C. elegans) *[42]. Phosphorylated AMPK then stimulates downstream signaling pathways that regulate organismal energy homeostasis.

**Fig. 2:**
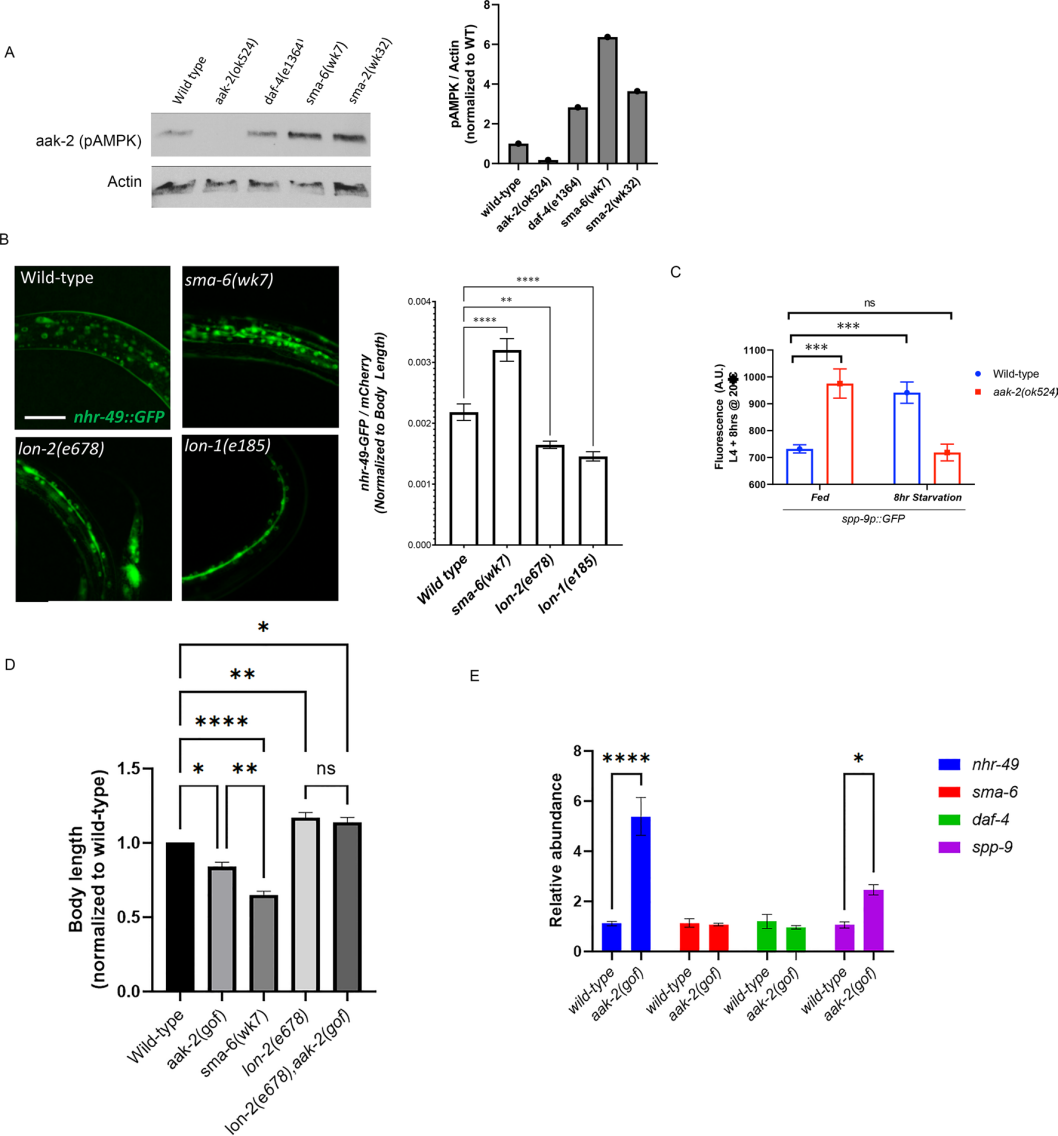
BMP signaling suppresses AMPK and AMPK negative feedback regulation of BMP in *C. elegans*. **A** Western blot analysis for pAMPK of *C. elegans* with BMP loss-of function *(lof)* mutants of BMPR2 *daf4(e1364)* and Smad transcription factors (sma-6 and sma-2) compared to wild-type worms. AMPK *lof* mutants aak-2 (ok524) were utilized as a control. Nematodes were grown at 20 °C until L4 stage. Each lane represents 25 animals. **B** Fluorescence images and quantification of *C. elegans* expressing the AMPK downstream reporter *nhr-49p::nhr-49::GFP* with genotypes harboring BMP *lof* (SMA-6) and *gof lon-1( e185),lon-2 ( e678)* (n = 30 animals). Images taken at L4 stage animals under well-fed conditions at 20 °C. Scale bar indicates 50 microns. **C** Fluorescence quantification of *C. elegans* expression of the BMP transcriptional reporter *spp-9::GFP* in AMPK wild-type and AMPK *lof* mutant *aak-2 (ok524)* in either well-fed animals or after 8 h of starvation (n = 30 animals). **D** Body-length measurements of indicated genotypes at L4 stage grown at 20 °C (n = 30 animals). **E** Relative quantification of mRNA levels of indicated genes in wild-type and AMPK *gof* mutant *aak-2* animals at L4 stage grown at 20 °C. All alleles are loss of function except for *aak-2(gof)*, which is a transgene that expresses a truncated, constitutively active kinase

Given that nutrient stress suppresses BMP signaling, we asked whether BMP signaling mutants themselves experienced nutrient stress and whether *aak-2* was activated in these animals. Western blot analysis identified that AMPK phosphorylation is enhanced in various BMP *loss-of-function (lof)* mutants of BMPR2 *daf4(e1364),* BMPR1 (SMA-6), and Smad transcription factor (SMA-2) compared to wild-type animals, even in well-fed conditions (Fig. 2A). AMPK *lof* mutants *aak-2 (ok524)* were utilized as a control. If AMPK were activated, we would expect that downstream targets of AMPK would also be upregulated in BMP mutants. One such target is the nuclear hormone receptor-49 (*nhr-49)*. We show that a *lof* BMP mutant SMA-6 exhibits increased expression of an *nhr-49p::nhr-49::GFP* transgene, whereas *gain-of-function (gof)* BMP mutants *lon-1(e185)* and *lon2 (e678)* exhibit decreased expression of this AMPK target (Fig. 2B). Taken together, these studies show that in *C. elegans,* BMP signaling inhibits AMPK activation during periods of ‘well-fed’ conditions, and that loss of signaling activates AMPK even with ample nutrition.

### AMPK negative feedback regulation of BMP signaling

We next examined whether feedback between AMPK and BMP signaling exists in *C. elegans*. The *lof* AMPK mutation *aak-2(ok542)* was crossed to BMP transcriptional reporter *spp-9p::GFP* to determine how BMP signaling is regulated when AMPK is absent. In fed conditions, we find that BMP signaling is suppressed in AMPK *lof* mutants, suggesting that AMPK promotes BMP signaling during nutrient-stable conditions (Fig. 2C). Importantly, we find that AMPK is required for the suppression of BMP signaling as *aak-2(ok542)* animals fail to increase *spp-9::GFP* expression under nutrient-deprived conditions when animals are starved for 8 h (Fig. 2C). Taken together, these results point a feedback regulation whereby BMP and AMPK signaling regulate each other to maintain energy homeostasis during periods of nutrient stress.

*C. elegans* BMP signaling governs growth and body-size [43]; BMP *lof* mutants have smaller body lengths compared with wild-type, whereas *gof* mutants exhibit longer body lengths. Our above results suggests that activation of AMPK inhibits BMP signaling; we wondered whether the ‘small’ body might be a result of activated AMPK in *lof* BMP mutants and vice versa in *gof* BMP mutants. We utilized an *aak-2(gof)* (AMPK) transgenic strain in which a truncated version of the AAK-2 protein has a gain-of-function phenotype [44]. We find that these *aak-2(gof)* mutants are smaller in body-size compared to wild-type animals (Fig. 2D). However, they fail to suppress the ‘long’ body phenotype of *lon-2(e678) gof* mutants. These results suggest that the regulation of body-size and energy homeostasis by BMP-AMPK cross-talk is uncoupled; other BMP-regulated genes are likely responsible for the body size regulation.

Finally, using a qRT-PCR approach, we show that AMPK inhibition of BMP signaling is through post-transcriptional mechanisms (Fig. 2E). In *aak-2(gof)* animals, *nhr-49* mRNA levels are upregulated as would be expected for a downstream target of AMPK activation. However, Type 1 BMP receptor (*sma-6)* and Type 2 BMP receptor (*daf-4)* mRNA levels are unchanged compared to wild-type, but *spp-9* mRNA levels are increased, showing that AMPK regulation of BMP signaling is post-transcriptional.

Taken together, these data show that nutrient-dependent energy homeostasis is regulated by BMP-AMPK cross-talk.

### BMP suppresses LKB1 in lung cancer cells

**Fig. 3:**
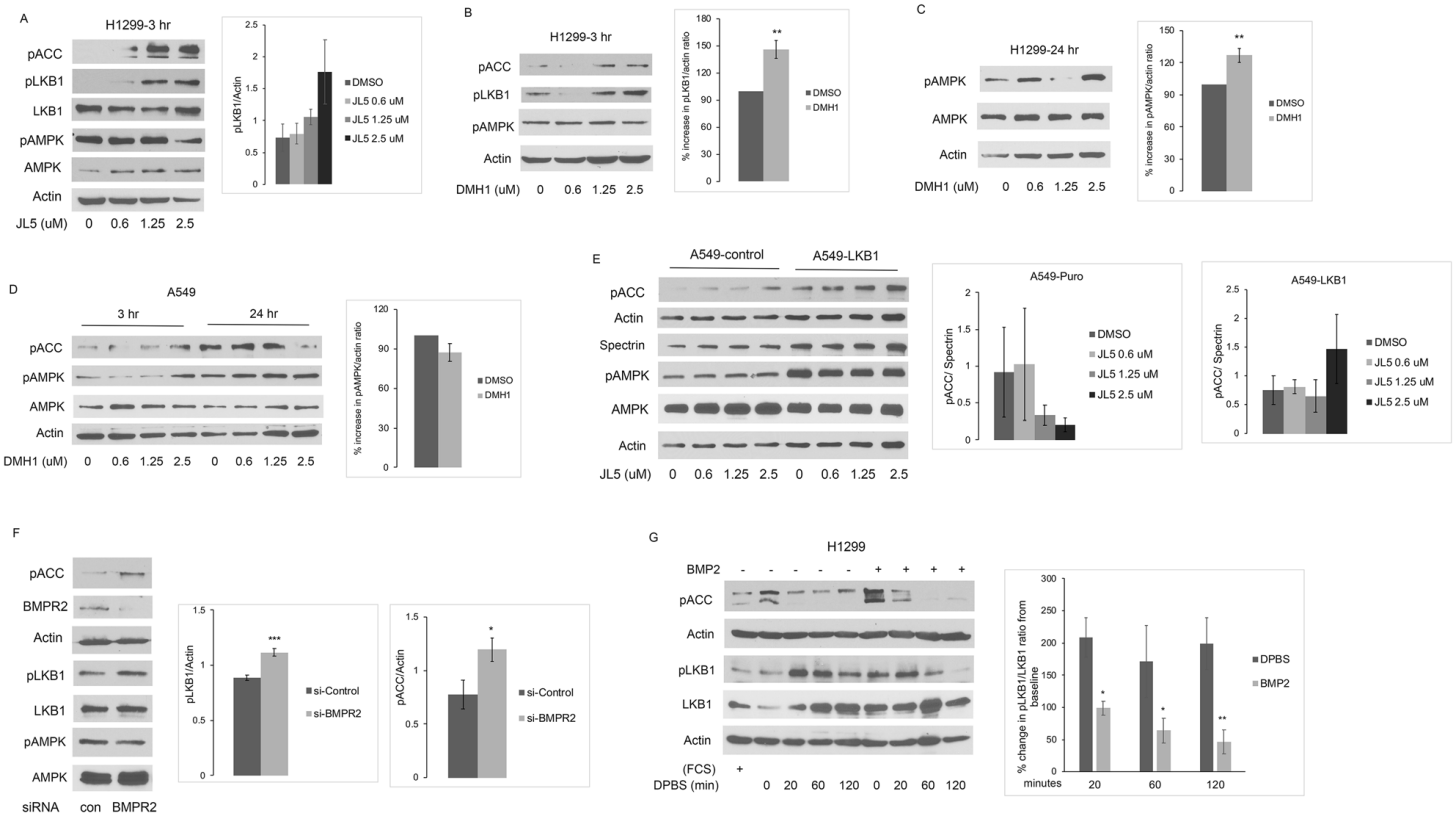
BMP suppresses LKB1 in lung cancer cells. **A**, **B** Western blot analysis of H1299 cells treated with JL5 or DMH1 demonstrating an increase in pACC and LKB1 at 3 h. Graph represents the mean pLKB1 expression normalized to Actin, n = 3. **B** Representative immunoblot. Mean percent increase in pLKB1/Actin ratio compared to control at 3 h following treatment with DMH1, n = 3. **C** Immunoblot of H1299 cells treated with DMH1 for 24 h. Mean percent increase in normalized pAMPK expression compared to control at 24 h following treatment with DMH1, n = 3 **D** Immunoblot of A549 cells treated with DMH1 for 3 and 24 h, (n = 3) demonstrating no significant change in pACC or pAMPK. The pLKB1, pAMPK, and Actin bands scanned were the 2.5 µM concentration except for one 1.25 µM peak value band obtained from H1299 cells treated with DMH1 for 3 h. **E** Western blot analysis of A549 cells stably expressing LKB1 and A549 control transfected cells treated with JL5 for 24 h. Both Actin and spectrin were used as loading controls. Graph represents mean pACC/spectin ratio, n = 2. **F** Immunoblot of H1299 cells following siRNA knockdown of BMPR2. Graph represents the mean of 4 independent studies. **G** Western blot analysis of starved H1299 cells treated with and without BMP2 ligand. Graph represent the mean change in pLKB1/pLKB1 ratio compared to control over time, n = 3

We examined whether BMP regulates AMPK/LKB1 signaling in lung cancer cells, which have aberrant expression of BMP signaling [45, 46]. Full activation of AMPK requires phosphorylation at Thr173, which in most cells is mediated by LKB1 [47]. H1299 cells treated the BMP receptor inhibitor JL5 [8], demonstrated an increased phosphorylation of the AMPK downstream target acetyl-CoA carboxylase (ACC) Ser79 within 3 h (Fig. 3A). LKB1 demonstrated an increase in phosphorylation at its Ser428 activation site at 3 h following treatment with JL5 without a change in phosphorylation of AMPK (Fig. 3A). This suggests that LKB1 activation occurs prior to that of AMPK following inhibition of BMP signaling. H1299 cells treated with the BMP receptor inhibitor DMH1 [48] also showed an increase in pACC Ser79 and pLKB1 Ser428 levels after 3 h but not in pAMPK levels (Fig. 3B). An increase in pAMPK at Thr173 was seen 24 h after treatment with DMH1 (Fig. 3C). A549 cells, which do not express LKB1, did not demonstrate an increase in expression in either pACC (Ser79) or pAMPK (Thr172) after 3 and 24 h of treatment with DMH1 (Fig. 3D). In A549-LKB1 cells, which are A549 cells with restored LKB1 from a stably expressed transgene, JL5 did not show an increase phosphorylation of AMPK at this time point but did enhance phosphorylation of ACC (Ser79) (Fig. 3E). These pharmacological studies suggest that BMP signaling suppresses AMPK signaling through the regulation of LKB1 in lung cancer cells.

BMPR2 siRNA was used to further evaluate the role of BMP signaling suppressing LKB1. BMPR2 siRNA knockdown in H1299 cells did not change the levels of pAMPK (Thr172) but did significantly increase pLKB1(Ser428) levels (Fig. 3F). BMPR2 knockdown also increased the phosphorylation of ACC (Ser79) (Fig. 3F). Moreover, in starved H1299 cells, BMP2 ligand significantly decreased pLKB1 (Ser428) and pACC (Ser79) levels over time while it increased in controls (Fig. 3G). These molecular studies complement our studies using BMPR2 inhibitors, further supporting the hypothesis that BMP signaling suppresses AMPK signaling.

### BMP signaling decreases TCA intermediates and non-essential amino acids in H1299 cells

**Fig. 4:**
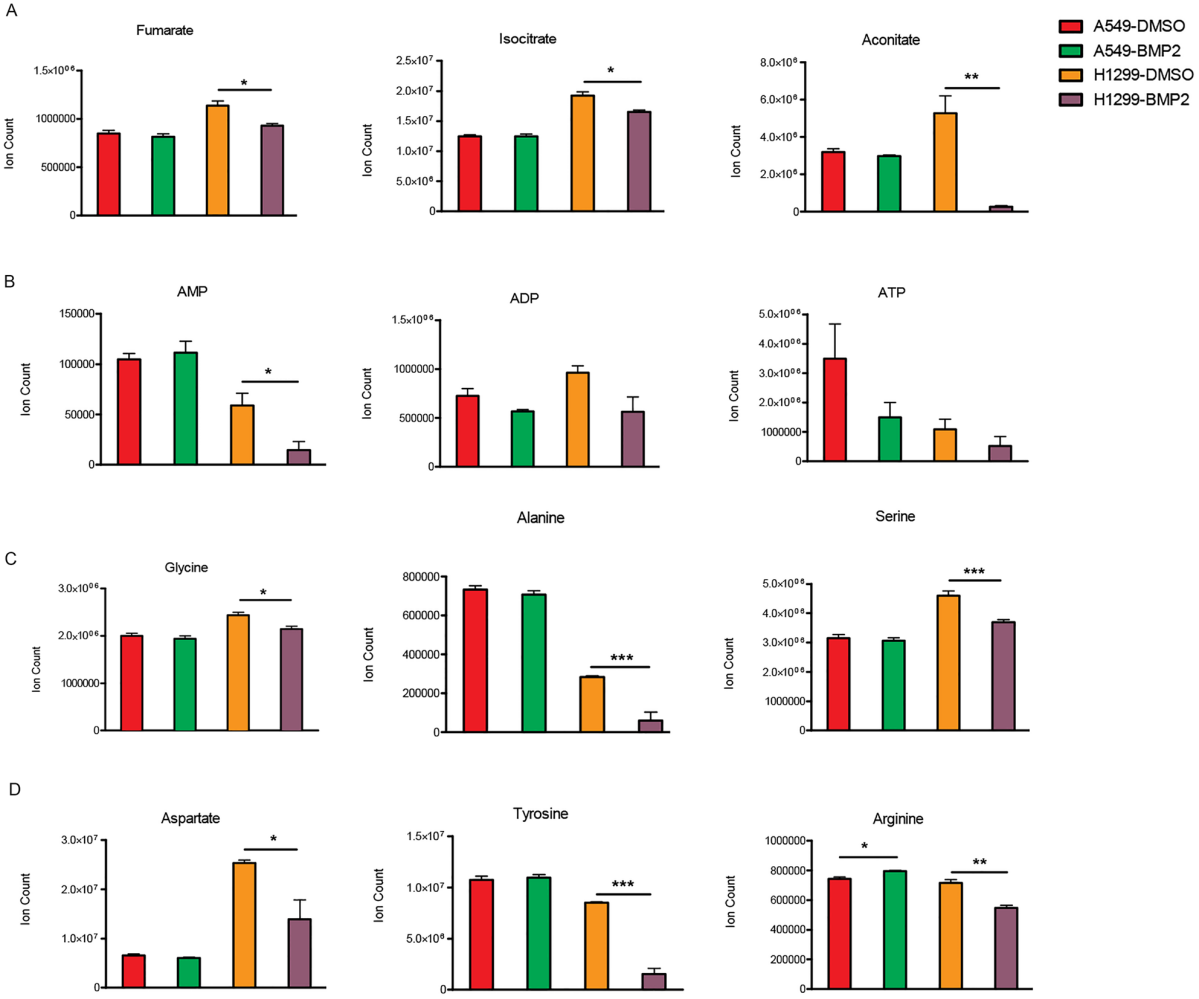
BMP2 ligand decreases expression of TCA cycle intermediates and non-essential amino acids. A549 and H1299 cells were treated with BMP2 ligand for 40 min and metabolomic analysis performed by LC-MC. Data represents mean of 3 experiments for **A** TCA cycle intermediates, **B** ATP intermediates, and **C**, **D** amino acids

Next, we examined whether BMP signaling regulated metabolism in lung cancer cells by examining changes in tricarboxylic acid cycle (TCA) intermediates and amino acids following treatment with BMP2 ligand. We examined TCA cycle intermediates and amino acids because suppression of AMPK would decrease TCA cycle activity [49], which would negatively effect production of essential amino acids [50, 51]. BMP2 treated H1299 but not A549 cells had significantly lower TCA intermediates compared to controls (Fig. 4A). BMP2 treated H1299 cells but not A549 cells also had significantly lower AMP levels (Fig. 4B). Although ADP and ATP levels trended lower in H1299 cells treated with BMP2, these differences did not reach statistical significance (Fig. 4B). Several non-essential amino acids derived from TCA cycle intermediates were also significantly decreased in BMP2 treated H1299 cells but not in A549 cells (Fig. C, D). These studies suggest that BMP2 has a suppressive effect on metabolic intermediates of the TCA cycle.

### AMPK suppresses BMP signaling in lung cancer cells

**Fig. 5:**
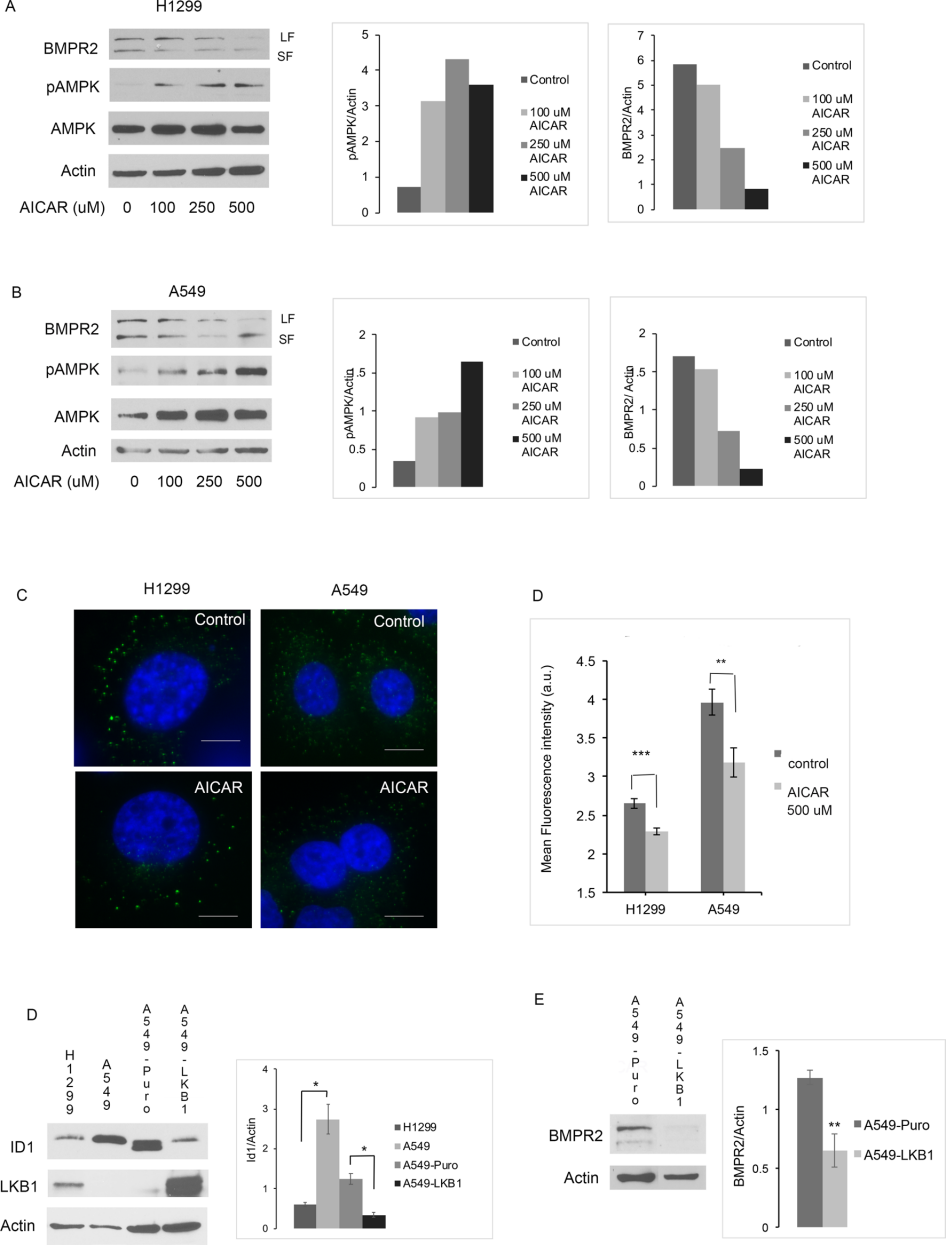
AMPK suppress BMP signaling in lung cancer cells. **A**, **B** Immunoblot showing long form (LF) and short form (SF) of lung cancer cells in regular media treated with increasing doses of the AMPK inhibitor AICAR for 24 h. AMPK expression was normalized to Actin. **C** Immunofluorescence imaging for BMPR2 at the cell surface of lung cancer cells treated with AICAR (500 μM) for 24 h. Graph of mean fluorescence intensity, n = 4. **D**, **E** Representative immunoblot of ID1 and BMPR2 in lung cancer cells with and without LKB1 expression. **D**, **E** quantification of normalized ID1 and BMPR2 expression, n = 3

Our prior studies showed that JL5 induces the mislocalization of BMPR2 from the plasma membrane to the cytosol [31, 52]. Since JL5 activates AMPK, we examined if AMPK affected the localization and expression of BMPR2. To activate AMPK, we used the allosteric activator AICAR. In both H1299 and A549 cells, AICAR caused a decrease protein expression of the long and short form of BMPR2 (Fig. 5A, B). Immunofluorescence imaging demonstrated in both H1299 and A549 that AICAR decreases the expression of BMPR2 at the plasma membrane (Fig. 5C). A549 cells express more ID1 then H1299 (Fig. 5D). A549 cells with LKB1 restored (A549-LKB1) have lower expression of ID1 (Fig. 5D) and BMPR2 (Fig. 5E). These studies suggest that AMPK decreases BMP signaling in lung cancer cells by downregulating BMPR2.

Taken together, these results show the conserved crosstalk between BMP and AMPK across phyla.

### BMP activation of PI3K requires BMPR2

**Fig. 6:**
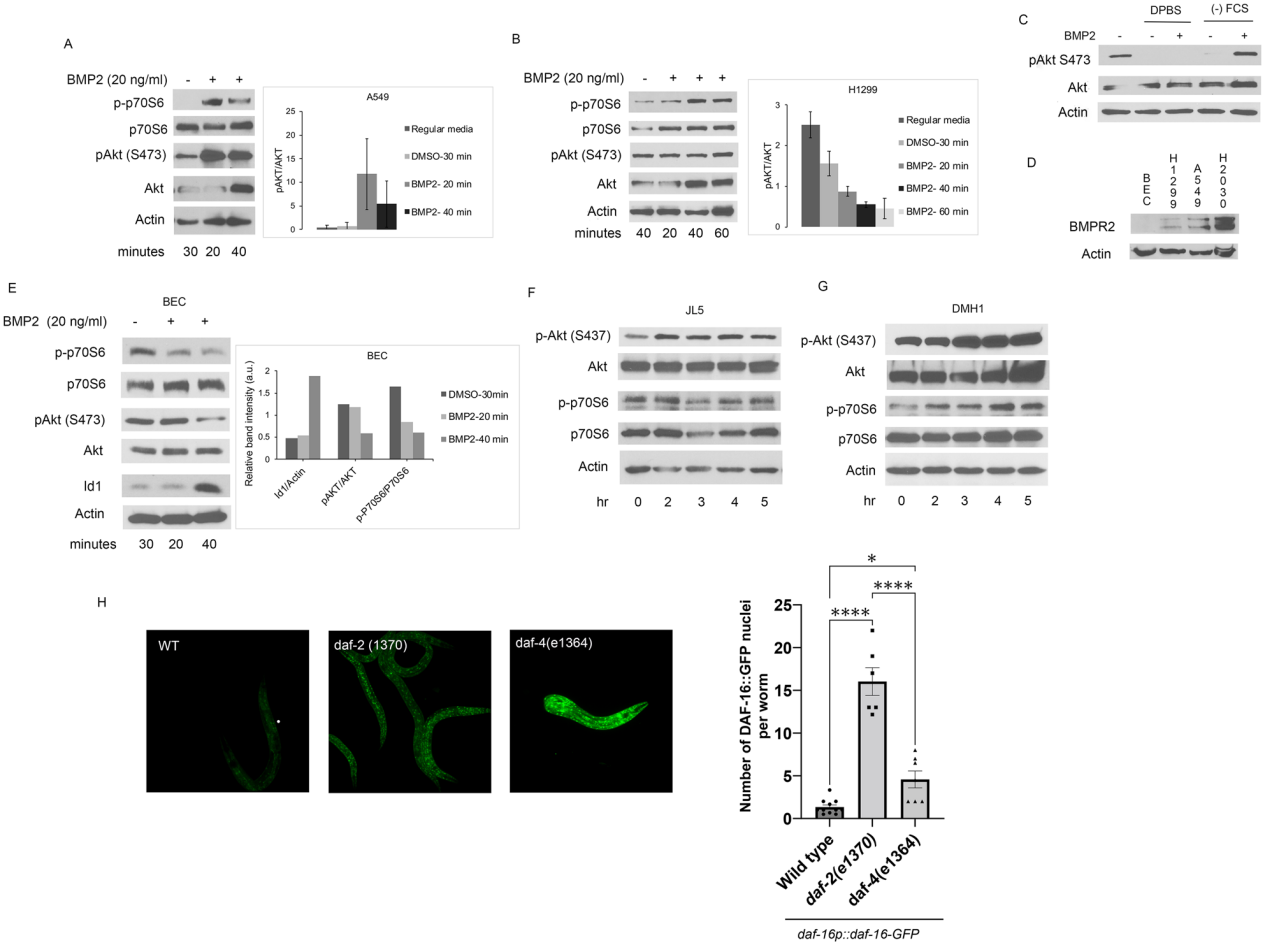
BMP activation of PI3k requires BMPR2. **A**, **B** Immunoblot of (**A**) A549 and (**B**) H1299 cells treated with BMP2 ligand with pAkt expression normalized to Akt, n = 2. **C** Immunoblot of A549 cell treated with BMP2 following starvation with DPBS or in regular media without FCS. **D** Immunoblot for BMPR2 expression of normal human bronchial epithelial cells (BEC) and lung cancer cell lines. **E** Immunoblot of BEC treated with BMP2 for 20 min. **F**, **G** Immunoblot of H1299 cells treated with BMP inhibitors JL5 2.5 μM or DMH1 2.5 μM for 24 h. **H** Fluorescence quantification of DAF-16::GFP positive nuclei in the indicated in insulin/DAF-2 *daf-2 (1370)* and BMPR2/DAF-4 *daf-4 (e1364)* at L4 stage grown at 20 °C

Our prior studies showed that BMP2 ligand activated mTORC1 (p-p70S6) signaling in lung cancer cells through phosphatidylinositol-3-kinase (PI3K) [22]. Whether BMP2 ligand signals through BMP type 1 or type 2 receptors in lung cancer cells has not been elucidated. In A549 cells, BMP2 activates both p-p70S6 and pAkt S473 (Fig. 6A). In the H1299 cells, BMP2 activated p70S6 but not Akt (Fig. 6B). During starvation there is a significant decrease in the expression of BMPR2 at cell surface (Fig. 1D). When A549 cells were starved for 20 min they were no longer responsive to BMP2 activation of Akt (Fig. 6C). By Western blot analysis, lung cancer cell lines express BMPR2 (Fig. 6D). BMPR2 expression was not detected in normal bronchial epithelial cells (BEC) (Fig. 6D). BMP2 did not active either p70S6 or Akt in BEC cells (Fig. 5E). BMP inhibitors of the type 1 receptors, JL5 and DMH1, did not inhibit either Akt or p70S6 in H1299 cells (Fig. 6F, G), suggesting the regulation is specific to type 2 receptors. These data suggest that BMPR2 is required for PI3K activation in lung cancer cells.

To assess if BMPR2 signaling through PI3K is conserved, we tested FoxO*/daf-*16 nuclear localization in *C. elegans* in BMPR2/*daf-4 lof* mutants*.* Insulin-like proteins signal through DAF-2 receptor activating PI3K/Akt signaling. The DAF-2*/*PI3K/Akt signaling pathway phosphorylates FoxO/DAF-16, inhibiting its entry into the nucleus thereby decreasing its activity [53]. As expected, wild-type animals show little-to-no DAF-16::GFP nuclear localization, whereas *daf-2(e1370) lof* mutants exhibit strong DAF-16::GFP nuclear localization (Fig. 6H). Interestingly, loss of BMPR2/*daf-4* significantly increases DAF-16::GFP nuclear localization compared to wild type, although this activation is not as strong as loss of insulin signaling (Fig. 6H). These studies suggest that BMPR2 regulation of PI3K is conserved in *C. elegans*.

### Insulin increase BMPR2 expression in lung cancer cells during starvation

**Fig. 7:**
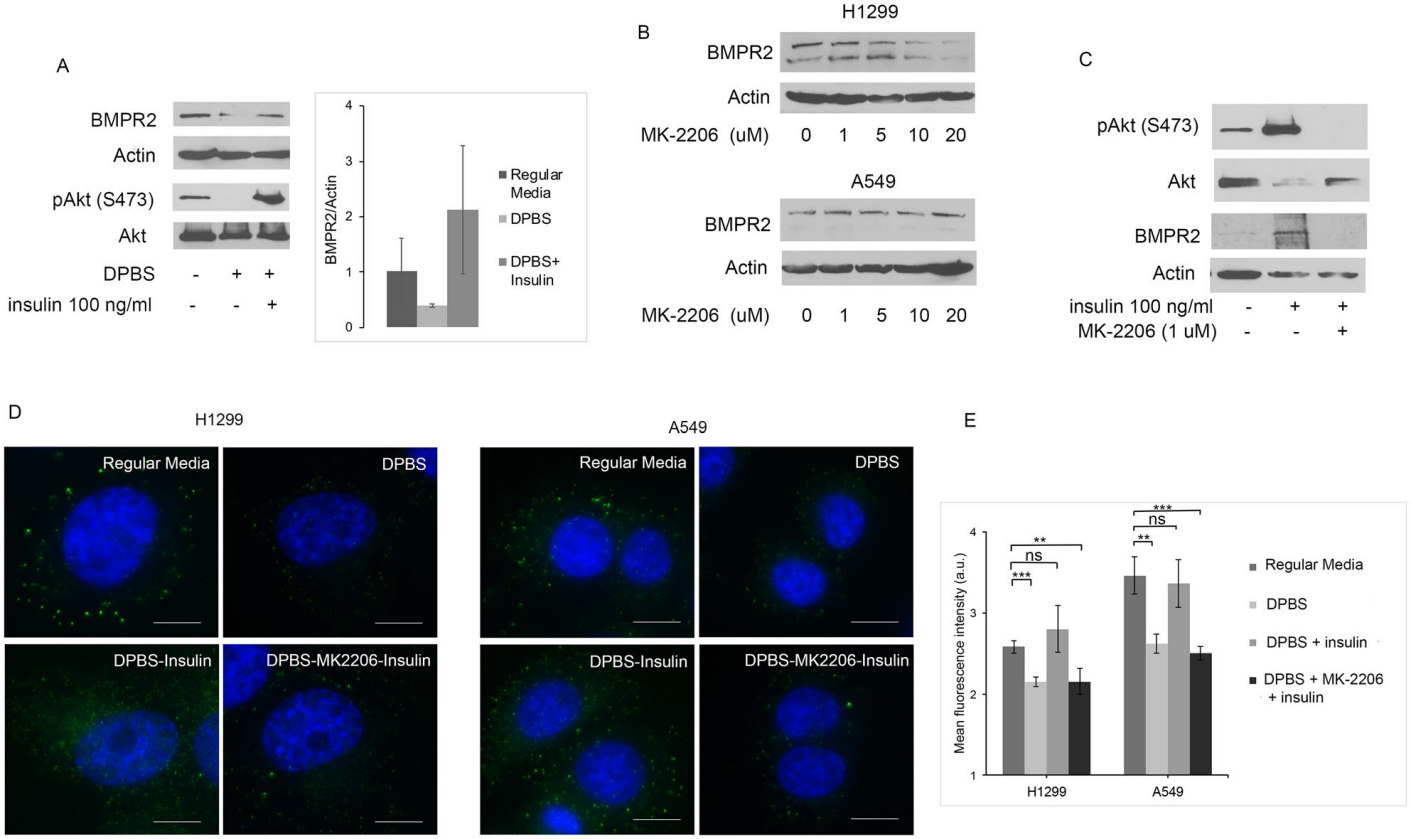
Akt increases BMPR2 expression during starvation. **A** Immunoblot of starved A549 cells or starved then treated with insulin for 60 min. BMPR2 expression was normalized to Actin, n = 2. **B** Immunoblot of lung cancer cells in regular media with FCS treated with Akt inhibitor MK-2206 for 24 h. **C** Immunoblot of starved A549 cells or starved then treated with insulin for 60 min with and without the Akt inhibitor MK-2206. **D** Immunofluorescence imaging for BMPR2 at the cell surface of starved cancer cells treated with insulin for 60 min with and without MK-2206. **E** Mean fluorescence of 4 experiments of starved cells treated with insulin for 60 min with and without MK-2206

Prior studies have shown that BMP7 enhances insulin signaling through PI3K, suggesting an interaction between BMPR2 and insulin signaling. We examined if insulin regulated BMPR2 in lung cancer cells. Unlike BMP2, insulin activated Akt in lung cancer cells during starvation (Fig. 7A). Insulin prevented the decrease in BMPR2 protein expression during starvation (Fig. 7A). To assess if there is feedback regulation from Akt, we used the specific Akt inhibitor MK-2206. In nutrient conditions, MK-2206 caused a decrease in protein expression of BMPR2 in H1299 cells but not A549 cells (Fig. 7B). In starved A549 cells, MK-2206 attenuated insulin’s increase in protein expression of BMPR2 and its activation of pAkt (Fig. 7C). Immunofluorescence imaging showed that insulin prevented the decreased expression of BMPR2 at plasma membrane during starvation in both H1299 and A549 cells (Fig. 7D, E). The inhibition of Akt with MK-2206 attenuated insulin’s increase in BMPR2 expression at the cell surface during starvation (Fig. 7D, E). These studies suggest that activated Akt causes a positive feedback regulation of BMPR2.

**Fig. 8:**
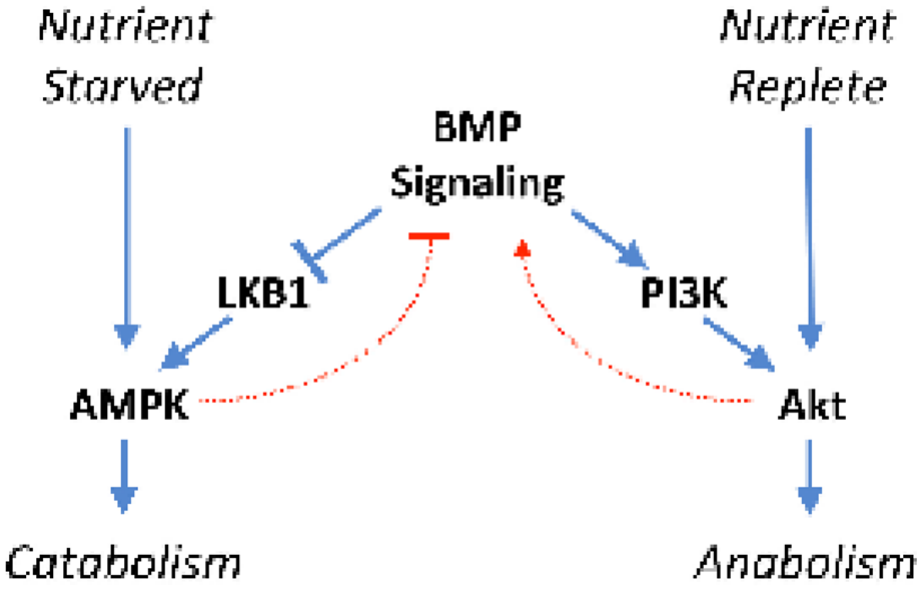
Schematic demonstrating integration of BMP signaling with anabolic and catabolic metabolism. Arrows indicate positive signaling interactions, whereas T-bars indicate negative signaling interactions. Solid blue arrows and T-bars indicate the primary effects of nutrients, nutrient starvation, and BMP signaling on metabolic signaling. BMP signaling promotes PI3K/Akt signaling and anabolism in response to abundant nutrients. By contrast, BMP signaling reduces AMPK signaling and catabolism in response to nutrient starvation. Dotted red arrows and T-bars indicate regulatory feedback by AMPK signaling and feedforward by PI3K/Akt signaling on BMP signaling

Fig. 8 demonstrates the integration of BMP with anabolic and catabolic signaling.

## Discussion

BMP ligand and receptor expression is increased in cancer and several other age-related diseases. Normally there is little signaling BMP in the mature lung [54]; however, BMP signaling is reactivated with inflammation and cancer [45, 54]. Although there is little to no BMP2 expression in normal lung tissue or benign tumors, BMP2 is highly expressed in 98% of non-small lung carcinoma (NSLC) [46]. During normal aging, BMP ligand expression increases tenfold in the dentate gyrus [3, 55]. BMP ligand expression is increased further in patients with Alzheimer’s disease [4]. The expression of BMP ligands and receptors are also increased in adipocytes in obese humans and mice [2, 17].

Aberrant BMP signaling is pathological. Studies in cancer show that BMP signaling enhances cell survival, migration, cancer stem cell self-renewal, and metastasis, and increased BMP expression is associated with a worse survival [8, 45, 56–60]. BMP induces the differentiation of neural stem cells into astrocytes and inhibits neuronal differentiation. Decreasing BMP signaling with the BMP inhibitor noggin or with exercise in mice promotes neurogenesis and improves cognition in mice [6, 61]. Adipose specific knockout of noggin enhances BMP activity, causing significant increase in white adipose tissue, fatty liver, and glucose intolerance in mice fed a high-fat diet [18]. BMP over-activity has also been shown to cause anemia of chronic disease [62] and heterotopic bone formation from a gain-of-function mutation in alk2 [63].

AMPK is a master regulator of catabolism, and like BMP, is phylogenetically conserved in metazoans [64]. AMPK catabolic responses are essential to maintain nutrient and energy availability. AMPK decreases anabolic signaling to conserve energy utilization, and activated AMPK also increases ATP production by stimulating mitochondrial biogenesis. The ability to activate AMPK has been shown to improved longevity across multiple species [65], while its suppression has been implicated in disease including diabetes, obesity, increased tumorigenicity, and a decrease in neurogenesis and synaptic activation [47, 66]. The mechanisms by which AMPK signaling is suppressed in disease are poorly understood.

We show in both *C. elegans* and lung cancer cells that BMP suppresses AMPK. Suppressing BMP signaling in lung cancer cells and *C. elegans* led to the activation of AMPK. These data demonstrate that pharmaceutical targeting of BMP receptors can induce AMPK activation. Inhibition of either BMP type 1 or BMP type 2 receptors led to the activation of AMPK. This suggests that inhibition of BMP type 1 receptors is required, but AMPK activation can be achieved by inhibiting BMPR2. Further studies are needed to determine whether it is best to target BMP type 1 or type 2 to promote activation of AMPK. Our studies suggest that BMP signaling inhibits AMPK by inhibiting LKB1. The regulation might be reciprocal, as prior studies have shown that LKB1 binds to BMP type 1 receptors, inhibiting their signaling by inducing degradation [67]. LKB1 has also been shown to phosphorylate Smad-4 on Thr^77^ inhibiting its binding to transcriptional targets of BMP and TGFβ [68]. Further studies are needed to determine the mechanism by which the interactions of BMP receptors and/or Smad-1/5 inhibit LKB1.

AMPK induced negative feedback regulation of BMP signaling in both lung cancer cells and *C. elegans*. Activated AMPK suppressed BMPR2 in both H1299 and A549 cells. Since A549 cells that do not express LKB1, it suggests that AMPK and not LKB1 mediates the downregulation of BMPR2. Although LKB1 expression may not be required to suppress BMPR2, it is required for full activation of AMPK. Therefore lung tumors with LKB1 *lof* would have less AMPK activity and less suppression of BMP signaling. LKB1 is mutated in 20–30% of NSCLC and is associated with a more aggressive phenotype. LKB1 mutated tumors having a less suppressive effect on BMP signaling in cancer is suggested by a prior report demonstrating that NSCLC with LKB1 *lof* had higher expression of BMP2 [67].

Prior studies have suggested that BMP signaling activates PI3K through BMPR2 independent of Smad activation. In mesenchymal progenitors, BMP2 mediates chemotaxis by inducing binding of BMPR2 to PI3K regulatory p55γ subunit [21]. Our studies in lung cancer cells and *C. elegans* are consistent with BMP signaling activating PI3K and downstream anabolic signaling pathways Akt and mTOR by BMPR2 Smad-independent mechanisms. BMP induction of anabolic signaling pathways was responsive to nutrient conditions. During starvation BMPR2 expression is decreased and lung cancer cells were no longer responsive to BMP2 ligand. Furthermore, Akt-mediated insulin signaling prevented the decrease in BMPR2 expression during starvation. Since a number of tyrosine kinases and oncogenic mutations, including K-ras, mediate cell growth through PI3K/Akt signaling, multiple oncogenes may utilize Akt to enhance BMPR2 signaling.

## Conclusion

We demonstrate that BMP signaling is integrated with the master regulators of anabolic and catabolic metabolism in cancer, which is conserved in *C. elegans*. Aberrant BMP signaling in lung cancer is growth promoting and has been shown to be pathological in other age-related diseases. These studies suggest the aberrant BMP signaling causes metabolic dysfunction, which has implications in causing pathological responses in cancer and potentially other age-related diseases.

## Data Availability

The datasets obtained and analyzed for this study will be made available from the corresponding author in a reasonable request.

## Abbreviations

BMP: Bone morphogenetic protein
BMPR2: Bone morphogenetic protein receptor 2
AIF: Apoptosis inducing factor
XIAP: X-linked inhibitor of apoptosis protein
TGFβ: Transforming growth factor beta
TAK1: Activated kinase 1
NSCLC: Non-small cell lung carcinomas
AMPK: AMP activated kinase
LKB1: Liver kinase B1
lof: Loss-of-function
gof: Gain-of-function
DPBS: Dulbecco’s phosphate-buffered saline
*nhr-49*: Nuclear hormone receptor-49
ACC: Acetyl-CoA carboxylase
kD: Kilodalton
PI3K: Phosphatidylinositol-3-kinase
